# Long-term 4CMenB Vaccine Effectiveness Against Gonococcal Infection at Four Years Post–Program Implementation: Observational Case–Control Study

**DOI:** 10.1093/ofid/ofae726

**Published:** 2024-12-13

**Authors:** Bing Wang, Lynne Giles, Prabha Andraweera, Mark McMillan, Rebecca Beazley, Jana Sisnowski, Sara Almond, Janine Mitchell, Noel Lally, Charlotte Bell, Louise Flood, James Ward, Helen Marshall

**Affiliations:** Vaccinology and Immunology Research Trials Unit, Women's and Children's Health Network, Adelaide, South Australia, Australia; Robinson Research Institute and Adelaide Medical School, The University of Adelaide, Adelaide, South Australia, Australia; Robinson Research Institute and Adelaide Medical School, The University of Adelaide, Adelaide, South Australia, Australia; School of Public Health, The University of Adelaide, Adelaide, South Australia, Australia; Vaccinology and Immunology Research Trials Unit, Women's and Children's Health Network, Adelaide, South Australia, Australia; Robinson Research Institute and Adelaide Medical School, The University of Adelaide, Adelaide, South Australia, Australia; Vaccinology and Immunology Research Trials Unit, Women's and Children's Health Network, Adelaide, South Australia, Australia; Robinson Research Institute and Adelaide Medical School, The University of Adelaide, Adelaide, South Australia, Australia; Communicable Disease Control Branch, SA Health, Adelaide, South Australia, Australia; Communicable Disease Control Branch, SA Health, Adelaide, South Australia, Australia; Communicable Disease Control Branch, SA Health, Adelaide, South Australia, Australia; Communicable Disease Control Branch, SA Health, Adelaide, South Australia, Australia; Communicable Disease Control Branch, SA Health, Adelaide, South Australia, Australia; Communicable Disease Control Branch, SA Health, Adelaide, South Australia, Australia; Communicable Disease Control Branch, SA Health, Adelaide, South Australia, Australia; UQ Poche Centre for Indigenous Health, University of Queensland, Brisbane, Queensland, Australia; Vaccinology and Immunology Research Trials Unit, Women's and Children's Health Network, Adelaide, South Australia, Australia; Robinson Research Institute and Adelaide Medical School, The University of Adelaide, Adelaide, South Australia, Australia

**Keywords:** 4CMenB, gonorrhea, meningococcal B vaccine, vaccine effectiveness, vaccine impact

## Abstract

**Background:**

A 4-component meningococcal B (4CMenB) vaccine program was introduced in adolescents in 2019 in South Australia. We aimed to evaluate long-term vaccine effectiveness (VE) and impact (VI) on gonococcal infection 4 years after implementation of the program.

**Methods:**

Disease notification data were provided by SA Health. VE was estimated as the reduction in the odds of infection using the screening and case–control methods. Time-to-event analyses assessed vaccine effect on reinfections. VI was estimated as incidence rate ratios (IRRs) using negative binomial regression models.

**Results:**

At 4 years after implementation of the program, 2-dose VE against gonococcal infection was 44.3% (95% CI, 35.1%–52.2%) using chlamydia patients as controls. Lower VE estimates were demonstrated in those >48 months post–4CMenB vaccination (26.2%; 95% CI, −2.6% to 47.0%) compared with those who had been vaccinated within ≤48 months (46.0%; 95% CI, 36.3%–54.2%). Slightly higher VE estimates were observed in females (48.7%; 95% CI, 36.9%–58.2%) compared with males (38.0%; 95% CI, 22.0%–50.7%). The risk of a second episode of gonococcal infection was lower in vaccinated gonococcal cases (adjusted hazard ratio, 0.682; 95% CI, 0.450–1.034). The adjusted IRR was 0.635 (95% CI, 0.421–0.957), with an observed 36.5% reduction in gonococcal infection.

**Conclusions:**

4CMenB demonstrates moderate effectiveness against gonococcal infection, with a lower VE estimate after 4 years postvaccination. 4CMenB may reduce the risk of recurrent gonococcal infection. The requirement for a booster dose and dosing interval warrants evaluation.

Invasive meningococcal disease is a potentially life-threatening infection caused by *Neisseria meningitidis* [1]. Serogroups A, B, C, W, X, and Y are the cause of most meningococcal cases globally. Two protein-based meningococcal serogroup B (MenB) vaccines are licensed for use worldwide: multicomponent vaccine 4CMenB (Bexsero, GSK) and MenB-FHbp (Trumenba, Pfizer)-containing lipidated FHbp [2].

Gonococcal infection caused by *N. gonorrhoeae* has become a major public health problem because of rapid development of antimicrobial resistance [3]. Prevention strategies for gonococcal infection encompass a range of measures aimed at reducing infection risk and transmission such as routine screening, early detection, and treatment. There is no licensed gonococcal vaccine against gonococcal infection [4].

Real-world evidence suggests that MenB vaccines with outer membrane vesicle (OMV)–based antigens offer moderate protection against gonococcal infection [5–10]. Very high genomic similarities exist between the 4CMenB vaccine antigens and *N. gonorrhoeae* protein sequences [11]. Proteins targeted by 4CMenB are present in both *N. meningitidi*s and *N. gonorrhoeae*. The high sequence identity for neisserial heparin binding antigen (NHBA) proteins in both *N. meningitidis* and *N. gonorrhoeae* suggests a protective effect induced by 4CMenB-generated antibodies [12]. However, evidence for waning immunity, protective effects against repeat infections and coinfections with other sexually transmitted infections (STIs), and differences in vaccine protection by gender is lacking or limited [5–8, 13–16].

4CMenB has been incorporated into numerous national or regional recommendations and infant programs, but it is infrequently extended to adolescents. In February 2019, a publicly funded 2-dose 4CMenB vaccine program was introduced in South Australia (SA) for senior school students (∼15 years of age), with a 1-time catch-up program extended to young adults under the age of 21 years. The ongoing program in SA provides an opportunity to assess the persistence of protection offered by 4CMenB vaccine in adolescents against gonococcal infection. This study aimed to assess the vaccine impact (VI) and vaccine effectiveness (VE) of 4CMenB against gonococcal infection 4 years after the implementation of the 4CMenB program in adolescents and young adults including VE against repeat infections.

## METHODS

### Study Design

The methods and statistical analysis plan were previously published [8, 16, 17]. A cohort study and a case–control study evaluated the VE and VI on gonococcal disease in SA 4 years after the 4CMenB vaccination program was implemented in February 2019.

### Data Source

Gonococcal and chlamydia notification data were provided by the Communicable Disease Control Branch (CDCB; SA Health) in SA. Data on laboratory confirmation, sociodemographic factors, sex at birth, and outcomes of all patients with gonococcal and/or chlamydia infections are collected by the CDCB in accordance with legislative requirements. Immunization records were extracted from the Australian Immunisation Register (AIR). Access to the South Australian records of the AIR was approved by the AIR Data Steward, Australian Government Department of Health. Probabilistic matching of MenB vaccination and notification records was undertaken using the first 2 letters of the first name, first 2 letters of the last name, and date of birth using the Stata module *reclink* with subsequent manual review (StataCorp). Gonococcal and chlamydia patients with matched MenB vaccination records were considered vaccinated; patients with no matches after manual review were considered not vaccinated. As all notifications from 2016 onward were reported with a unique patient identification number, person-level data were utilized in the VE analysis. This allowed for the identification of repeat gonococcal and chlamydia infections and gonococcal and chlamydia coinfections. Infection-level data were employed in the VI analysis.

### Vaccine Impact

VI was calculated by comparing gonococcal cases reported in the 8 pre–vaccine program years with those reported in the 4 program years postintroduction (from February 1, 2019, to January 31, 2023) for all adolescents and young adults aged 15 to 26 years at the time of notification or estimated disease onset. Negative binomial regression models were fit to estimate VI on gonococcal notifications. The age cohorts were categorized according to the estimation of MenB vaccine coverage.

### Vaccine Effectiveness

To evaluate VE against gonococcal infection, the case–control method [7] was used. Chlamydia controls were matched to gonococcal cases by date of birth ±28 days, with matching by replacement. Gonococcal cases were included if they were aged ≤19 years on February 1, 2019 (born after February 1, 1999), aged ≥15 years during each program year, and had an infection notification date between February 1, 2019, and January 31, 2023. Due to uncertainty regarding the timing of post–vaccination immunity against gonococcal infection, and as gonococcal infection can be asymptomatic and present months before screening or diagnosis, vaccine doses were counted only if disease onset occurred ≥3 months after the vaccine dose. In this study, exposure was defined as receiving either a partial (1-dose) or full (2-dose) vaccine series, with the first or second dose administered at least 3 months before disease onset. The model was adjusted for sex at birth, age, and socioeconomic status as potential confounding factors. Gonococcal and chlamydia patients with repeat diagnoses were included in analyses. The first recorded notification after the MenB vaccination was used to define the index infection in all vaccinated individuals with repeat infection, which was reported with a minimum interval of 30 days since the last notification. Any occurrence of gonococcal and chlamydia infections within 7 days was regarded as a coinfection. Logistic regression with a random effect for person and robust estimation of standard errors was used. The VE estimates using the screening method [18, 19] and case–control method with 20 AIR controls [20] were also assessed as defined in the protocol. For each gonococcal case, up to 20 controls were randomly selected from a de-identified AIR data set, matched by birth date within ±28 days. A control's vaccine dose was considered valid if administered at least 3 months before the case's laboratory confirmation date. Conditional logistic regression was performed for analysis. For the screening method, VE was calculated among vaccine-eligible 15–19-year-olds with confirmed gonococcal infection, over 4 years in SA, using the formula VE = 1 – [PCV/(1-PCV)]/[PPV/(1-PPV)], where PCV is the proportion of vaccinated cases and PPV is the age-matched population vaccine coverage at the time of case notification. Logistic regression was used in the screening method, with case vaccination status as the outcome variable, fitting only a constant and including an offset for the log odds of the matched vaccine coverage. VE was calculated as 1 minus the odds ratio (OR) estimated from the model. VE confidence limits were calculated as 1 minus the OR confidence limits.

Subgroup analyses were undertaken to explore the duration of protection against gonococcal infection, the effect of sex at birth, and coinfection with chlamydia. To assess VE over time, gonococcal and chlamydia infections that occurred 3–48 months or >48 months after the date of the second dose were categorized and analyzed separately. VE was estimated separately in females and males. Because gonococcal coinfection with chlamydia is common and the VE might differ between gonococcal infection with or without chlamydia coinfection, we estimated VE by (1) comparing cases coinfected with gonorrhea and chlamydia to controls who had chlamydia only and (2) excluding all coinfections and then comparing cases who had gonococcal infection only with controls who had chlamydia only.

Multivariable Cox proportional hazards regression models were fit to estimate the adjusted hazard ratio (HR) and 95% CI for repeat gonococcal infection in vaccinated vs unvaccinated gonococcal cases. The analyses were adjusted for sex and socioeconomic status with stratification of age to satisfy assumption of proportional hazards. These covariates were selected based on scientific relevance and their potential relationship with both the exposure and outcome. The Akaike Information Criterion (AIC) was used to balance improvements in model fit with complexity. Grambsch-Therneau tests were performed to evaluate the proportional hazards assumption in the Cox proportional hazards regression models. Separate analyses were performed for 2 outcome measures: a single failure event, which was the first repeat gonococcal infection, and multiple failure events, encompassing all repeat gonococcal infections. All analyses were conducted using Stata, version 18 (StataCorp).

The study was approved by the SA Department for Health and Wellbeing Human Research Ethics Committee (HREC No.: 2022/HRE00308), Aboriginal Health Research Ethics Committee (AHREC Protocol#: 04-22-1027), and Human Research Ethics Committee of NT Health and Menzies School of Health Research (NT HREC No.: 2023–4662).

### Patient Consent

This study included only data collected as part of routine notifications and immunization records. As only de-identified surveillance data were provided and analyzed, a waiver of consent was justified based on the National Statement on Ethical Conduct in Human Research in Australia and approved by the above-mentioned ethics committees.

## RESULTS

At 4 years after implementation of the 4CMenB vaccination program, 1-dose (77.3%, 16885/21847) and 2-dose (68.8%, 15029/21847) coverage was highest in adolescents born in 2005 (∼15 years of age).

### Vaccine Impact on Gonococcal Infection

At 4 years following the implementation of the state adolescent program, the fitted negative binomial regression model showed a 36.5% decrease (adjusted IRR, 0.635; 95% CI, 0.421–0.957) in gonococcal notifications among the adolescents aged 15–17 years with high vaccine uptake (Table 1).

**Table 1.** Incidence of Gonococcal Infection Before and After Implementation of the Adolescent Ongoing and Adolescent/Young Adult Catch-up Programs

	Average Annual No. of Gonococcal Notifications (Annual Incidence of Gonococcal Notifications [per 100 000 Population])						Average Annual No. of Gonococcal Notifications (Annual Incidence of Gonococcal Notifications [per 100 000 Population])						Adjusted Incidence Rate Ratio^a^ (95% CI) [Postvaccination Period vs Prevaccination Period]					
	Feb 2011–Jan 2012	Feb 2012–Jan 2013	Feb 2013–Jan 2014	Feb 2014–Jan 2015	Feb 2015–Jan 2016	Feb 2016–Jan 2017	Feb 2017–Jan 2018	Feb 2018–Jan 2019	Feb 2019–Jan 2020	Feb 2020–Jan 2021	Feb 2021–Jan 2022	Feb 2022–Jan 2023	Feb 2017–Jan 2018	Feb 2018–Jan 2019	Feb 2019–Jan 2020	Feb 2020–Jan 2021	Feb 2021–Jan 2022	Feb 2022–Jan 2023
15–17 y	48.5 (78.769) [Prevaccination period 2011–2017]						45.5 (75.693) [‘B Part of It’ study period^b^]		65 (106.915) [Postvaccination period 2019–2023]				0.618 (0.410–0.933) *P* = .022 [‘B Part of It’ study period^b^]		0.635 (0.421–0.957) *P* = .030 [Postvaccination period 2019–2023]			
18–20 y	110.5 (167.762) [Prevaccination period 2011–2019]								178.25 9 (281.327) [Postvaccination period 2019–2023]						0.891 (0.615–1.291) *P* = .542 [Postvaccination period 2019–2023]			
21–26 y^c^	291 (208.632) [Prevaccination period 2011–2020]									401.7 (286.929) [Postvaccination period 2020–2023]						0.783 (0.530–1.156) *P* = .218 [Postvaccination period 2020–2023]		

^a^These incidence rate ratios were adjusted according to changes in the incidence of gonococcal notifications in all 3 age cohorts of adolescents/young adults who were not eligible to receive the free 4CMenB vaccine.

^b^The impact of the “B Part of It” study was accounted for in the adjusted model, as this was a state-wide vaccination initiative. The 4CMenB vaccinations were administered in 237 schools and campuses across metropolitan Adelaide and rural and remote South Australia, representing 96.1% of enrolled senior school students.

^c^The cohort of young adults, ranging from age 21 to 26 years, did not meet the criteria for the state MenB immunization program in 2019. As those who were under 21 years old in 2019 advanced in age and transitioned into the 21–26 age group in 2020, this cohort became partially eligible for the state MenB immunization program post-2020 with low vaccine coverage.

### Vaccine Effectiveness Against Gonococcal Infection

There were 1295 gonococcal and 7317 chlamydia notifications reported in 1058 gonococcal and 5569 chlamydia patients during the 4 program years. Five gonococcal patients and 22 chlamydia patients received >2 vaccine doses, and 1 patient with gonococcal infection could not be matched with any chlamydia patients because of their young age; these patients were all subsequently excluded from analysis. The primary analysis thus included 1052 gonococcal patients matched with 5544 chlamydia patients. Of the 1052 gonococcal patients, 149 experienced recurring gonococcal infection (69 females, 80 males), and 320 had coinfection with chlamydia (168 females, 152 males). There was a higher proportion of females among chlamydia controls (66.8%) compared with gonococcal cases (51.5%; χ² = 89.571; df = 1; *P* < .001). A higher percentage of gonococcal cases were from low–socioeconomic status areas (62.5%; χ² = 91.563; df = 2; *P* < .001) and identified as Aboriginal (33.0%; χ² = 541.545; df = 1; *P* < .001) compared with chlamydia controls (47.0% from low–socioeconomic status areas and 6.9% identifying as Aboriginal). Among chlamydia controls, 41.1% had received the 2-dose 4CMenB, and 6.1% had received the 1-dose 4CMenB. A lower percentage of gonococcal cases were vaccinated, with 26.5% receiving 2 doses and 4.6% receiving 1 dose of 4CMenB. Among gonococcal cases, Aboriginal individuals (12.4%, 43/346; χ² = 53.931; df = 2; *P* < .001) and those residing in low–socioeconomic status areas (20.8%, 136/653; χ² = 41.185; df = 4; *P* < .001) had a lower percentage of 2-dose 4CMenB vaccination compared with non-Aboriginal individuals (33.6%, 236/703) and those residing in mid– and high–socioeconomic status areas (30.0%, 67/225, in mid–; 44.9%, 75/167, in high–socioeconomic status areas).

Using chlamydia controls, the estimated VE was 44.3% (95% CI, 35.1%–52.2%) for people who received 2 doses in comparison with people who were unvaccinated (Table 2). By using 20 AIR controls, the VE estimate was 59.8% (95% CI, 53.2%–65.4%) for 2 doses. Applying the screening method, the VE was 59.7% (95% CI, 53.3%–65.2%) for 2 doses.

**Table 2.** Adjusted ORs and VE for Two-Dose 4CMenB Against Gonococcal Infection in Primary, Subgroup, and Sensitivity Analyses^a^

	Adjusted OR (95%CI)	Vaccine Effectiveness (95% CI), %
Primary and subgroup analyses: duration of protection		
1) Vaccinated (>3 mo) with 2-dose 4CMenB vs unvaccinated^b^ (primary analysis)	0.557 (0.478–0.649) *P* < .001	44.3 (35.1–52.2)
2) Vaccinated (within 3–48 mo) with 2-dose 4CMenB vs unvaccinated^c^	0.540 (0.458–0.637) *P* < .001	46.0 (36.3–54.2)
3) Vaccinated (>48 mo) with 2-dose 4CMenB vs unvaccinated^d^	0.738 (0.530–1.026) *P* = .071	26.2 (−2.6 to 47.0)
Subgroup analysis: sex at birth		
1) Vaccinated (>3 mo) with 2-dose 4CMenB vs unvaccinated in females^e^	0.513 (0.418–0.631) *P* < .001	48.7 (36.9–58.2)
2) Vaccinated (>3 mo) with 2-dose 4CMenB vs unvaccinated in males^f^	0.620 (0.493–0.780) *P* < .001	38.0 (22.0–50.7)
Subgroup analysis: gonococcal cases coinfecting with chlamydia		
1) Vaccinated with 2-dose 4CMenB vs unvaccinated in gonococcal cases with coinfection and chlamydia controls without coinfection^g^	0.566 (0.438–0.729) *P* < .001	43.4 (27.1–56.2)
2) Vaccinated with 2-dose 4CMenB vs unvaccinated in gonococcal cases without coinfection and chlamydia controls without coinfection^h^	0.553 (0.462–0.662) *P* < .001	44.7 (33.8–53.8)
Sensitivity analysis: a shorter interval (>1 mo) between disease diagnosis and second dose^b^		
Vaccinated with 2-dose 4CMenB vs unvaccinated^i^	0.552 (0.473–0.643) *P* < .001	44.8 (35.7–52.7)
Sensitivity analysis: a longer interval (>6 mo) between disease diagnosis and second dose^b^		
Vaccinated with 2-dose 4CMenB vs unvaccinated^j^	0.558 (0.479–0.650) *P* < .001	44.3 (35.0–52.1)

^a^Cases and controls with any missing data were excluded from the analyses.

^b^Primary analysis: 1045 gonococcal cases were included, and 5492 chlamydia controls were matched to gonococcal cases with replacement (148 729 chlamydia controls were included after matching).

^c^999 gonococcal cases were included, and 5064 chlamydia controls were matched to gonococcal cases with replacement (129 420 chlamydia controls were included after matching).

^d^813 gonococcal cases were included, and 3648 chlamydia controls were matched to gonococcal cases with replacement (83 939 chlamydia controls were included after matching).

^e^540 gonococcal cases were included, and 3680 chlamydia controls were matched to gonococcal cases with replacement (48 351 chlamydia controls were included after matching).

^f^500 gonococcal cases were included, and 1803 chlamydia controls were matched to gonococcal cases with replacement (26 272 chlamydia controls were included after matching).

^g^319 gonococcal cases were included, and 5455 chlamydia controls were matched to gonococcal cases with replacement (44 026 chlamydia controls were included after matching).

^h^726 gonococcal cases were included, and 5485 chlamydia controls were matched to gonococcal cases with replacement (104 753 chlamydia controls were included after matching).

^i^1045 gonococcal cases were included, and 5492 chlamydia controls were matched to gonococcal cases with replacement (148 779 chlamydia controls were included after matching).

^j^1045 gonococcal cases were included, and 5492 chlamydia controls were matched to gonococcal cases with replacement (148 729 chlamydia controls were included after matching).

In the subgroup analyses, a lower 2-dose VE estimate was demonstrated after 48 months postvaccination (26.2%; 95% CI, −2.6% to 47.0%), and the 2-dose VE estimate was higher within 3–48 months postvaccination (46.0%; 95% CI, 36.3%–54.2%) (Table 2). The VE estimate was higher in females (48.7%; 95% CI, 36.9%–58.2%) compared with males (38.0%; 95% CI, 22.0%–50.7%). However, the difference between females and males was not significant when incorporating an interaction term of sex at birth and the number of doses into the primary analysis model (adjusted OR, 0.917; 95% CI, 0.675–1.248; *P* = .583). The VE estimates were comparable when considering both the exclusion (44.7%; 95% CI, 33.8%–53.8%) and inclusion (44.3%; 95% CI, 35.1%–52.2%) of gonococcal cases with coinfection. Furthermore, the VE estimate did not vary substantially when comparing gonococcal cases with (43.4%; 95% CI, 27.1%–56.2%) vs without coinfection with chlamydia controls. In the sensitivity analyses, changing the vaccine eligibility interval from 3 months to either 1 or 6 months did not significantly change the VE results.

In multivariable Cox proportional hazards models adjusting for potential confounders, the adjusted HR for incidence of a first repeat gonococcal infection comparing fully vaccinated with unvaccinated cases was 0.682 (95% CI, 0.450–1.034; *P* = .072). The adjusted HR for all repeat gonococcal infections was 0.942 (95% CI, 0.619–1.432; *P* = .778).

## DISCUSSION

Our findings contribute to the real-world evidence regarding the vaccine impact and effectiveness of 4CMenB against gonococcal infection. Our study specifically examined the impact of sex, coinfection with chlamydia, recurrent gonococcal infection, and duration of protection. The extensive program implemented in SA stands as a unique initiative within both the Australian and global landscapes, offering invaluable insights to inform ongoing and future 4CMenB vaccine programs and policy decisions.

In the NZ studies, the VE of 3-dose MeNZB against gonococcal infection was estimated as 31% (95% CI, 21%–39%) [7] and the VE against hospitalization caused by gonococcal infection was 24% (95% CI, 1%–42%) [15]. In addition to the OMV vaccine (MeNZB) in NZ, 4CMenB comprises 3 recombinant proteins (NHBA, FHbp, and NadA), which exhibit variable shared characteristics with *N. gonorrhoeae*. Notably, NadA is absent in all studied strains, and FHbp is not expressed on the surface. The NHBA antigen in 4CMenB is surface-exposed in *N. gonorrhoeae*. Antibodies induced by 4CMenB have been reported to recognize the gonococcal homolog of NHBA [12]. In the United States, VE of 4CMenB vaccine against gonococcal infection in people aged 16–23 years was assessed in New York City and Philadelphia. The estimated 2-dose VE against gonococcal infection was 40% (95% CI, 23%–53%) [5], and MenB-FHbp was shown to be ineffective against gonococcal infection (adjusted prevalence ratio, 0.97; 95% CI, 0.79–1.19) [21]. In Southern California, gonococcal infection rates were compared among recipients of 4CMenB vs MenACWY to avoid the potential bias of willingness to seek health care in vaccinees. The risk of gonococcal infection was 46% lower among recipients of 4CMenB vs MenACWY (adjusted HR, 0.54; 95% CI, 0.34–0.86), with chlamydia rates being similar between vaccine groups [6]. In Italy, 2-dose VE was estimated to be 44% (95% CI, 6%–65%) against gonococcal infection among people with HIV with a previous diagnosis of STI [22]. Among university students in the United States, 4CMenB demonstrated an effectiveness of 47% (95% CI, 13%–68%) in preventing gonococcal infection in recipients aged 18 to 29 years [23]. Our 2- [8] and 3-year evaluation [16] results showed that the 2-dose VE estimates (at 2 years: 32.7%; 95% CI, 8.3%–50.6%; at 3 years: 33.2%; 95% CI, 15.9%–47.0%) were lower than the 4-year evaluation estimate. This variation could be attributed to the use of a larger sample size in the 4-year evaluation with improved precision around the estimate or to the added benefits of reduced transmission, which would subsequently decrease the incidence. In a prospective, randomized trial of the combination of postexposure prophylaxis with doxycycline and 4CMenB vaccination (DOXYVAC), the final analysis revealed that 4CMenB did not significantly reduce the incidence of gonococcal infection (adjusted HR, 0.78; 95% CI, 0.60–1.0) because several gonorrhea episodes had accidentally not been included in the interim analysis [14]. Because this study was prematurely discontinued after interim analysis [24] and before reaching the projected sample size, it might be underpowered to demonstrate moderate vaccine efficacy against gonorrhea.

In line with findings from the MeNZB study conducted in NZ with a VE of 36% (95% CI, 22%–48%) in women and 25% (95% CI, 11%–36%) in men, our study similarly observed a difference in VE between males and females that was not significant. Up to 40% of urogenital *N. gonorrhoeae* infections in women may be asymptomatic [25]. If left untreated or poorly managed, these infections can result in severe consequences such as pelvic inflammatory disease, ectopic pregnancy, and infertility [3]. Preventative measures are particularly important for protecting women from complications associated with untreated or inadequately managed gonococcal infection.

Other studies have not examined repeat gonococcal infection. Prior studies have demonstrated the ability of *N. gonorrhoeae* to evade or resist host immune responses during natural infection. If 4CMenB can provide immunity against gonococcal infection for a duration of up to 4 years, it may offer protection against recurrent gonococcal infection. We estimated a risk of a single repeat infection, where disease burden is high and the vaccine may be less effective. Further vaccine research is required to investigate correlates of protection for gonococcal infection.

The VE estimates of MeNZB over time implied that the vaccine protection gradually diminishes after a certain period [7]. The difference in duration of protection may relate to the addition of OMPs in 4CMenB compared with MeNZB. The duration of protection of a theoretical vaccine had a significant effect on the prevalence of *N. gonorrhoeae* in the epidemiological simulation model [13]. This 4-year evaluation estimated that the VE against gonococcal infection wanes after 48 months (44.3% within 48 months; 26.2% after 48 months). The relatively small number of people receiving 4CMenB over 48 months previously may still be underpowered to identify a significant VE estimate. As a booster dose has not been recommended for adolescents and young adults who have finished a primary course of 2-dose MenB vaccines, further evaluation and consideration regarding the potential need for booster vaccination is necessary.

We found that gonococcal coinfection with chlamydia did not result in a significant variation in the VE estimates of 4CMenB in the subgroup analysis, which contrasts with the 4CMenB and MeNZB studies conducted in the United States and New Zealand [7]. The number and proportion of coinfections among patients in our study were notably lower in comparison with those observed in the US and NZ studies. This variance may suggest significant differences in the underlying epidemiological and sexual risk factors between our study population and those in the US and NZ studies. Changing the vaccine eligibility interval between the diagnosis and MenB vaccination from 3 months to 1 or 6 months did not vary VE results substantially, which may indicate that an adequate immune response had already developed 1 month after MenB vaccination. The US study, which employed a 30-day cutoff and excluded 4CMenB doses administered <30 days before STIs, reported a similar VE estimate of 40% against gonococcal infection [5].

The DOXYVAC study in France reported that doxycycline reduced gonococcal infection in MSM on HIV PrEP [14]. However, 2 studies conducted among MSM in France and women in Kenya did not observe a significant decrease in gonococcal infection [26, 27]. There are also concerns about long-term effects of doxycycline, particularly its potential negative impact on human microbiomes and antimicrobial resistance [28]. As there are currently no vaccines available with high effectiveness against gonococcal infection, 4CMenB may offer an available option for reducing the associated disease burden.

A key strength of this study lies in its utilization of a highly vaccinated cohort involved in an ongoing population-level program, in conjunction with systematic documentation of vaccines administered across the entire Australian population. The integration of enhanced disease surveillance, linked to vaccination records reported to the national immunization database, facilitates timely assessments of vaccine impact and effectiveness. Our VE analyses employed a triangulation approach, incorporating 2 distinct control sources and a screening method. This approach allowed us to examine methodological bias and validate our evaluation results. The utilization of individual-level data in the vaccine effectiveness analysis allowed us to explore the variability in VE associated with repeat infection and coinfection, while also enabling the follow-up of these patients over the 4-year period.

Our study has certain limitations. The potential impact of 4CMenB on incidence of gonococcal infection may be confounded by the COVID-19 pandemic, as public health restrictions during this period could have reduced the risk of gonococcal infection. COVID-19 and related restrictions might have influenced sexual behaviors and health care access, potentially affecting infection rates and vaccine accessibility during the study period. Although fluctuations in health care access and reporting of sexual behaviors might have occurred during lockdown periods, chlamydia patients were used as our control group, meaning that any COVID-related impacts would have affected both cases and controls concurrently. Older adolescents may have a higher exposure risk. To account for this, age was included as a covariate in our models to adjust for potential confounding effects on vaccine effectiveness estimates. Therefore, these factors were unlikely to have significantly altered our primary findings on vaccine effectiveness. Furthermore, most of the eligible vaccine cohort had received 4CMenB <4 years earlier, with some uncertainty regarding the estimate of immunity waning after 4 years. Changes in time-varying confounders, particularly behavioral factors, may have influenced our findings on vaccine effectiveness over time. Our study was unable to measure these behavioral factors directly; therefore, future research that includes detailed longitudinal behavioral data would help to further clarify these results. A long-term VE study is needed to investigate the duration of immunity against gonococcal infection. In addition to observational studies assessing VE and VI, randomized controlled trials are crucial for directly evaluating the efficacy of OMV-based MenB vaccines in reducing both gonococcal infection and transmission.

Our study revealed a notable decrease in gonococcal notifications and demonstrated sustained cross-protection against gonococcal infection in adolescents and young adults. Ongoing surveillance and evaluation are crucial for obtaining more conclusive evidence concerning potential waning immunity and in special groups. This information will guide policy-makers in the need for booster vaccination programs and consideration of the use of 4CMenB across a broader population to provide protection against 2 diseases with a single vaccine.

## References

[1] Wang B, Santoreneos R, Giles L, Afzali HHA, Marshall H. Case fatality rates of invasive meningococcal disease by serogroup and age: a systematic review and meta-analysis. Vaccine 2019; 37:2768–82. 30987851 10.1016/j.vaccine.2019.04.020

[2] Borrow R, Taha MK, Giuliani MM, Pizza M, Banzhoff A, Bekkat-Berkani R. Methods to evaluate serogroup B meningococcal vaccines: from predictions to real-world evidence. J Infect 2020; 81:862–72. 32745637 10.1016/j.jinf.2020.07.034

[3] Unemo M, Seifert HS, Hook EW 3rd, Hawkes S, Ndowa F, Dillon JR. Gonorrhoea. Nat Rev Dis Primers 2019; 5:79. 31754194 10.1038/s41572-019-0128-6

[4] Lyu Y, Choong A, Chow EPF, et al Vaccine value profile for *Neisseria gonorrhoeae*. Vaccine 2024; 42(19S1):S42–69. 38123397 10.1016/j.vaccine.2023.01.053 PMC11169088

[5] Abara WE, Bernstein KT, Lewis FMT, et al Effectiveness of a serogroup B outer membrane vesicle meningococcal vaccine against gonorrhoea: a retrospective observational study. Lancet Infect Dis 2022; 22:1021–9. 35427490 10.1016/S1473-3099(21)00812-4 PMC10227473

[6] Bruxvoort KJ, Lewnard JA, Chen LH, et al Prevention of *Neisseria gonorrhoeae* with meningococcal B vaccine: a matched cohort study in Southern California. Clin Infect Dis 2023; 76:e1341–9. 35642527 10.1093/cid/ciac436

[7] Petousis-Harris H, Paynter J, Morgan J, et al Effectiveness of a group B outer membrane vesicle meningococcal vaccine against gonorrhoea in New Zealand: a retrospective case-control study. Lancet 2017; 390:1603–10. 28705462 10.1016/S0140-6736(17)31449-6

[8] Wang B, Giles L, Andraweera P, et al Effectiveness and impact of the 4CMenB vaccine against invasive serogroup B meningococcal disease and gonorrhoea in an infant, child, and adolescent programme: an observational cohort and case-control study. Lancet Infect Dis 2022; 22:1011–20. 35427492 10.1016/S1473-3099(21)00754-4

[9] Whelan J, Kløvstad H, Haugen IL, Holle MR, Storsaeter J. Ecologic study of meningococcal B vaccine and *Neisseria gonorrhoeae* infection, Norway. Emerg Infect Dis 2016; 22:1137–9. 27191543 10.3201/eid2206.151093 PMC4880101

[10] Diaz LMR, Gonzalez ML, Cuello M, et al VA-MENGOC-BC vaccination induces serum and mucosal anti *Neisseria gonorrhoeae* immune responses and reduces the incidence of gonorrhea. Pediatr Infect Dis J 2021; 40:375–81. 33591079 10.1097/INF.0000000000003047

[11] Marjuki H, Topaz N, Joseph SJ, et al Genetic similarity of gonococcal homologs to meningococcal outer membrane proteins of serogroup B vaccine. mBio 2019; 10:e01668–19. 31506309 10.1128/mBio.01668-19 PMC6737241

[12] Semchenko EA, Tan A, Borrow R, Seib KL. The serogroup B meningococcal vaccine bexsero elicits antibodies to *Neisseria gonorrhoeae*. Clin Infect Dis 2019; 69:1101–11. 30551148 10.1093/cid/ciy1061 PMC6743822

[13] Craig AP, Gray RT, Edwards JL, et al The potential impact of vaccination on the prevalence of gonorrhea. Vaccine 2015; 33:4520–5. 26192351 10.1016/j.vaccine.2015.07.015 PMC4743649

[14] Molina JM, Bercot B, Assoumou L, et al Doxycycline prophylaxis and meningococcal group B vaccine to prevent bacterial sexually transmitted infections in France (ANRS 174 DOXYVAC): a multicentre, open-label, randomised trial with a 2 × 2 factorial design. Lancet Infect Dis 2024; 24:1093–104. 38797183 10.1016/S1473-3099(24)00236-6

[15] Paynter J, Goodyear-Smith F, Morgan J, Saxton P, Black S, Petousis-Harris H. Effectiveness of a group B outer membrane vesicle meningococcal vaccine in preventing hospitalization from gonorrhea in New Zealand: a retrospective cohort study. Vaccines (Basel) 2019; 7:5. 30621260 10.3390/vaccines7010005 PMC6466174

[16] Wang B, Giles L, Andraweera P, et al 4CMenB sustained vaccine effectiveness against invasive meningococcal B disease and gonorrhoea at three years post programme implementation. J Infect 2023; 87:95–102. 37268223 10.1016/j.jinf.2023.05.021

[17] Marshall HS, Andraweera PH, Wang B, et al Evaluating the effectiveness of the 4CMenB vaccine against invasive meningococcal disease and gonorrhoea in an infant, child and adolescent program: protocol. Hum Vaccin Immunother 2021; 17:1450–4. 33428528 10.1080/21645515.2020.1827614 PMC8078704

[18] Ladhani SN, Andrews N, Parikh SR, et al Vaccination of infants with meningococcal group B vaccine (4CMenB) in England. N Engl J Med 2020; 382:309–17. 31971676 10.1056/NEJMoa1901229

[19] Parikh SR, Andrews NJ, Beebeejaun K, et al Effectiveness and impact of a reduced infant schedule of 4CMenB vaccine against group B meningococcal disease in England: a national observational cohort study. Lancet 2016; 388:2775–82. 28100432 10.1016/S0140-6736(16)31921-3

[20] Quinn HE, Gidding HF, Marshall HS, et al Varicella vaccine effectiveness over 10 years in Australia; moderate protection from 1-dose program. J Infect 2019; 78:220–5. 30528868 10.1016/j.jinf.2018.11.009

[21] Abara WE, Bernstein KT, Lewis FMT, et al Healthy vaccinee bias and MenB-FHbp vaccine effectiveness against gonorrhea. Sex Transm Dis 2023; 50:e8–10. 36863060 10.1097/OLQ.0000000000001793 PMC10175191

[22] Raccagni AR, Galli L, Spagnuolo V, et al Meningococcus B vaccination effectiveness against *Neisseria gonorrhoeae* infection in people living with HIV: a case-control study. Sex Transm Dis 2023; 50:247–51. 36728240 10.1097/OLQ.0000000000001771

[23] Robison SG, Leman RF. Association of group B meningococcal vaccine receipt with reduced gonorrhea incidence among university students. JAMA Netw Open 2023; 6:e2331742. 37651146 10.1001/jamanetworkopen.2023.31742 PMC10472183

[24] Molina J, Bercot B, Assoumou L, Michele I, Rubenstein E, Pialoux G. ANRS 174 DOXYVAC: an open-label randomized trial to prevent STIs in MSM on PrEP. Paper presented at: 30th Conference on Retroviruses and Opportunistic Infections (CROI); February 2–22, **2023**; Seattle, WA, USA.

[25] McCormack WM, Stumacher RJ, Johnson K, Donner A. Clinical spectrum of gonococcal infection in women. Lancet 1977; 1:1182–5. 68279 10.1016/s0140-6736(77)92720-9

[26] Stewart J, Oware K, Donnell D, et al Doxycycline prophylaxis to prevent sexually transmitted infections in women. N Engl J Med 2023; 389:2331–40. 38118022 10.1056/NEJMoa2304007 PMC10805625

[27] Molina JM, Charreau I, Chidiac C, et al Post-exposure prophylaxis with doxycycline to prevent sexually transmitted infections in men who have sex with men: an open-label randomised substudy of the ANRS IPERGAY trial. Lancet Infect Dis 2018; 18:308–17. 29229440 10.1016/S1473-3099(17)30725-9

[28] Kong FYS, Kenyon C, Unemo M. Important considerations regarding the widespread use of doxycycline chemoprophylaxis against sexually transmitted infections. J Antimicrob Chemother 2023; 78:1561–8. 37129293 10.1093/jac/dkad129 PMC10577522

